# High Content Image-Based Screening of a Protease Inhibitor Library Reveals Compounds Broadly Active against Rift Valley Fever Virus and Other Highly Pathogenic RNA Viruses

**DOI:** 10.1371/journal.pntd.0003095

**Published:** 2014-08-21

**Authors:** Rajini Mudhasani, Krishna P. Kota, Cary Retterer, Julie P. Tran, Chris A. Whitehouse, Sina Bavari

**Affiliations:** Molecular and Translational Sciences Division, United States Army Medical Research Institute of Infectious Diseases, Frederick, Maryland, United States of America; National Center for Advancing Translational Sciences, United States of America

## Abstract

High content image-based screening was developed as an approach to test a protease inhibitor small molecule library for antiviral activity against Rift Valley fever virus (RVFV) and to determine their mechanism of action. RVFV is the causative agent of severe disease of humans and animals throughout Africa and the Arabian Peninsula. Of the 849 compounds screened, 34 compounds exhibited ≥50% inhibition against RVFV. All of the hit compounds could be classified into 4 distinct groups based on their unique chemical backbone. Some of the compounds also showed broad antiviral activity against several highly pathogenic RNA viruses including Ebola, Marburg, Venezuela equine encephalitis, and Lassa viruses. Four hit compounds (C795-0925, D011-2120, F694-1532 and G202-0362), which were most active against RVFV and showed broad-spectrum antiviral activity, were selected for further evaluation for their cytotoxicity, dose response profile, and mode of action using classical virological methods and high-content imaging analysis. Time-of-addition assays in RVFV infections suggested that D011-2120 and G202-0362 targeted virus egress, while C795-0925 and F694-1532 inhibited virus replication. We showed that D011-2120 exhibited its antiviral effects by blocking microtubule polymerization, thereby disrupting the Golgi complex and inhibiting viral trafficking to the plasma membrane during virus egress. While G202-0362 also affected virus egress, it appears to do so by a different mechanism, namely by blocking virus budding from the *trans* Golgi. F694-1532 inhibited viral replication, but also appeared to inhibit overall cellular gene expression. However, G202-0362 and C795-0925 did not alter any of the morphological features that we examined and thus may prove to be good candidates for antiviral drug development. Overall this work demonstrates that high-content image analysis can be used to screen chemical libraries for new antivirals and to determine their mechanism of action and any possible deleterious effects on host cellular biology.

## Author Summary

Rift Valley fever (RVF) is an arthropod-borne viral zoonosis that occurs in large parts of sub-Saharan and North Africa and in 2000 emerged outside the African continent for the first time, raising concerns that it could further expand its geographical range. The disease in humans can result in encephalitis or hemorrhagic fever and in ruminants often results in abortion in pregnant females. Due to the lack of a licensed and commercially available vaccine, efforts to discover effective antiviral drugs are underway. Drug discovery using high content image-based screening is an effective tool that has been successfully used to identify new drugs. In this study, we developed an image-based assay to identify compounds active against RVF virus and other highly pathogenic human viruses. We demonstrated the usefulness of our image-based high content assay in identifying potential RVF antivirals by screening a small subset of chemical compounds for inhibition of RVF virus in a human cell line (HeLa) and partially characterized their mechanism of action within infected cells. The methods we developed in this study will be useful in discovering new effective drugs to combat Rift Valley fever.

## Introduction

Many RNA viruses are highly pathogenic to humans and can cause hemorrhagic fever and/or encephalitis. Among these, Rift Valley fever virus (RVFV), a member of the genus *Phlebovirus* (family *Bunyaviridae*), causes Rift Valley fever (RVF) that severely affects human and livestock throughout Africa and the Arabian Peninsula [1], [2]. In humans, RVF is characterized by a flu-like illness that occasionally progresses to hemorrhagic fever, encephalitis, or ocular disease with a significant death rate [3]. In ruminants, RVF is characterized by a high rate of abortion in pregnant females (80–100%), as well as high mortality in younger animals [4]. RVF is a mosquito-borne disease transmitted by the bite of various species of mosquitoes or through direct contact with infected animals [5], [6]. Similar to RVFV, Lake Victoria marburgvirus (MARV), Zaire ebolavirus (ZEBOV), Lassa virus (LASV) and Venezuelan equine encephalomyelitis virus (VEEV) are highly pathogenic viruses and classified as National Institute of Allergy and Infectious Disease (NIAID) category A priority pathogens. Marburgviruses and ebolaviruses, members of the family *Filoviridae*, as well as LASV, members of the family *Arenaviridae*, cause severe hemorrhagic fevers in humans and nonhuman primates with extraordinarily high case-fatality rates (reviewed in [7], [8]). VEEV, an alpha virus and member of *Togaviridae* family, causes severe encephalitis in horses and humans (reviewed in [9]). RVFV, as with other highly pathogenic RNA viruses, including EBOV, MARV, VEEV and LASV, cause severe disease in many developing countries that already suffer from fragile economies and health care infrastructures. There is currently no U.S. Food and Drug Administration (FDA) approved therapeutic or prophylactic treatments for any of these agents, thus there is an urgent need for research to develop effective new drugs and vaccines to combat these diseases.

Recent advancements in high content image (HCI)-based screening (HCS) technologies have contributed greatly to increasing the efficiency of the drug discovery process. HCS utilizes automated high-speed, high-resolution microscopy and image analysis to measure morphological changes in the cells in a quantitative and high-throughput manner [10]. Most importantly, HCI-based analysis enables simultaneous measurement of multiple features of cellular biology that are relevant to therapeutic and cytotoxic characteristics of potential antiviral compounds. As a result, HCI-based analyses not only allows for rapid screening of compounds, but can provide early insights into their cytotoxicity and mode of action, thereby facilitating the decision-making processes that govern the progression from a candidate compound to a successful antiviral drug.

RVFV is an enveloped spherical virus with containing a has a tri-segmented, single-stranded RNA genome, which encodes for the RNA-dependent RNA polymerase (RdRp), envelope glycoproteins (Gn/Gc), nucelocapsid proteins (N) and non-structural proteins including NSs and NSm [11]. Virus entry into cells is mediated by the binding of the envelope glycoproteins (Gn/Gc) to an unknown cell surface receptor which mediates virus endocytosis. Acidification of the virus-containing endocytotic vesicle promotes virus-host membrane fusion and results in the release of the encapsidated genome and RdRp into the cytoplasm, where transcription and replication of the viral genome occurs [12]. The glycoproteins Gn and Gc form a heteromeric complex and localize in steady-state to the Golgi apparatus due to a Golgi localization signal on Gn [6], [10], [13]. Multiple interactions between the glycoporteins, the encapsidated viral genome, and remaining structural proteins are believed to cause a change in membrane curvature leading to virus budding into the Golgi lumen [14]–[16]. Although the exact mechanism is not known, based on knowledge from other bunyaviruses, it is possible that the release of the virus-filled vesicles from the Golgi and its subsequent fusion with cell plasma membrane, releases mature virions into extracellular medium.

During the past two years, small molecule libraries containing compounds related to currently marketed drugs with known targets; mostly targeting kinases, phosphatases, or G protein coupled receptors; have been used with high throughput or HCI-based screening for antiviral drug discovery [17]–[19]. However these studies in which HCI analysis was used primarily focused on assay development and optimization specifically for determining the antiviral activity of the compounds and were not applied for the determination of the compound's mechanism of action [18], [19]. Proteases constitute one of the largest drug target enzyme families, and their promise has been demonstrated by several successful drugs on the market, including the angiotensin converting enzyme inhibitors and anti-HIV protease inhibitors (reviewed in [20]). Many other drugs targeting key proteases involved in various diseases are predicted to reach the market in near future. Despite their success as drugs in the marketplace, to our knowledge, no studies using a protease inhibitor library and HCI-based analysis to screen for their antiviral activities have been previously reported.

Here we describe the development and application of HCS to test a protease inhibitor library of 849 small molecules for antiviral activity against multiple pathogenic RNA viruses and to determine their mechanism of action for RVFV.

## Methods

### Cells and Viruses

HeLa, Vero and Vero E6 cells were obtained from the American Type Culture Collection (ATCC) and maintained in Dulbecco's Modified Eagle Medium (Life Technologies, USA) supplemented with 10% fetal bovine serum (FBS, Life Technologies, USA). Human small airway epithelial cells (HSAECs) (Lonza, USA) were maintained under humidified conditions at 37°C, 5% CO_2_ in Ham's F12 medium supplemented with nonessential amino acids, pyruvate, β-mercaptoethanol and 10% fetal calf serum (FCS). RVFV strains ZH548/MP-12 (MP-12) and ZH501 were obtained from The Salk Institute's Government Services Division and from Dr. Michael Turell (USAMRIID), respectively. RVFV strain MP12 was derived from a virulent strain of RVFV (ZH548) and is highly attenuated in its virulence due to several nucleotide mutations in its genome, however, it retains its sensitivity to IFN-α [16], [21] and can be handled safety under biosafety level (BSL)-2 laboratory conditions. ZH501 is the wild-type strain of RVFV and is fully virulent, requiring to be handled under BSL-3 laboratory conditions. VEEV (1CSH3), LASV (Josiah), EBOV (Zaire) and MARV (Ci67) were obtained from the USAMRIID collection and were propagated in Vero cells. Virus-containing supernatant was clarified by centrifugation at 12,000×*g* for 30 min prior to storage at −80°C. All virus stock titers were determined by plaque assay on Vero E6 cells as previously described [22].

### Virus Infections

Work with infectious filoviruses or arenaviruses were performed under BSL-4 laboratory conditions. RVFV ZH501 and VEEV infections were performed in a BSL-3 lab, and RVFV MP12 infections were performed under BSL-2 conditions. HeLa cells were infected with virus at the indicated MOIs. Inocula were removed after 1 h, unless stated otherwise, washed one time with 1× PBS, and replaced with the same amount of fresh medium. Infection was allowed to proceed for a specific duration of time, as indicated for each experiment. At the end of the incubation time, culture supernatants were collect to determine the virus yield by plaque assay. Alternatively, virus-infected cells were fixed in 10% neutral buffered Formalin (Sigma) for 3 days for BSL-3/4 viruses or 15 min for BSL-2 viruses.

### Compound Library and Reference Reagents

ChemDiv was the source of the Serine/Cysteine Protease (SCP) inhibitor library used in this study. ChemDiv built the library using a variety of computational programs to qualify a small molecule as a potential SCP ligand (reviewed in [23]). Basically, their method applied a set of pre-selected descriptors for encoding the molecular structures, and a trained neural network to qualify the molecules as potential SCP ligands. The molecular requirements were profiled by using available databases of active SCP and non-SCP-active agents. The library was composed of 849 compounds in a 96-well plate format and was used in both primary and secondary screening assays. For subsequent mechanism of action studies, compounds were reordered (ChemDiv, USA) at least three different times and tested independently to ensure consistency across batches. Reference reagents included human interferon-α2a (IFN-α) (PBL Interferon Source, USA), ribavirin (Sigma), Brefeldin A (cat # B-7450, Life Technologies) and Nocodazole (cat # M1404, Sigma). All compounds from the ChemDiv protease inhibitor library and ribavirin were prepared in 100% dimethyl sulfoxide (DMSO, Sigma-Aldrich, USA) at 8 mM stock solution and stored at −20°C. IFN-α was prepared in Dulbecco's phosphate-buffered saline (DPBS, Sigma-Aldrich, USA) containing 5% FBS.

### Immunofluorescence Detection of RVFV-Infected Cells

An immunofluorescence assay (IFA) was used to visualize RVFV-infected cells. Briefly, cells were fixed with Formalin (10%) for 15 min at room temperature. To permeabilize cells, Formalin-fixed cells were treated with 0.1% (v/v) Triton-X 100 for 15 min at room temperature. RVFV-infected cells were by visualized by staining with 4D4 mAb prepared in blocking buffer containing 3% BSA/PBS. The mAb 4D4 binds to the Gn portion of the RVFV glycoprotein and was purified from the hybridomas at USAMRIID. The following antibodies, R3-1D8-1-1a, 1A4A-1, 6D8-1, MBG II 9G4-1 and L52-161-6 were used to detect, RVFV nucleocapsid (N), VEE envelope 2 (E2) protein, EBOV glycoprotein (GP), MARV GP and Lassa virus GP, respectively. Antibodies for cellular protein detection including Histone 3 phospho Serine 10 (cat #9710; Cell Signalling), alpha-Tubulin (cat #T9026; Sigma) and Alexa Fluor 488 phalloidin (cat # A12379; Life Technologies) were used at 1∶1000 dilution according to vendors' specifications. Cell nuclei and cytoplasm were labeled with Hoechst 33342 (Life Technologies, USA) and HCS CellMask Red or Deep Red (Life Technologies, USA), respectively, at a 1∶10,000 dilution. Golgi apparatus was visualized using CellLight Golgi-GFP (Life Technologies, USA), a modified baculovirus expressing a fusion construct of a Golgi marker and green florescent protein. Alexa 488-conjugated goat anti-mouse secondary antibody, Alexa 568-conjugated goat anti-rabbit antibody or Alexa 647-conjugated goat anti-rabbit (1∶1,000; Life Technologies, USA) were used to visualize primary antibodies.

### Primary Screening of the ChemDiv Protease Inhibitor Library

The ChemDiv protease inhibitor library was screened against RVFV strain MP12 at 20 µM in 0.5% (v/v) DMSO. Screening was performed in triplicates wells of a 96-well plate. HeLa cells were treated with compound for 2 h prior to virus infection. After virus infection, cells were washed with PBS 1×, and media containing the compound was added back to the cells and remained for the duration of the infection (24 h). To test the potential broad-spectrum antiviral activity of the primary hit compounds, HeLa cells were treated with the compounds in the same way as in the primary screen and infected with multiple RNA viruses as described below. The multiplicity of infection (MOI) and duration of infection for each virus were optimized to achieve an infection rate of 40–60% and were as follows: RVFV ZH501 (MOI = 1 for 24 h), EBOV (MOI = 3 for 48 h), MARV (MOI = 3 for 48 h), LASV (MO1 = 1 for 24 h), and VEEV (MOI = 0.5 for 20 h). Mock-infected HeLa cells were considered as negative controls, and wells in which the cells were infected with virus, but treated with 0.5% DMSO were considered as positive controls. Compound activity based on percent inhibition and cell toxicity was assessed by IFA and image analysis as described below. Data were plotted in Excel to generate a heat map using “color scales” in the conditional format menu. Scatter plot distribution was generated using TIBCO Spotfire 4.5.0 (TIBCO Software, USA).

### Image Analysis

Confocal images were collected using a Leica TCS-SP5 confocal/multiphoton microscope. High-content quantitative imaging data were acquired and analyzed on an Opera confocal reader (model 3842 [Quadruple Excitation High Sensitivity] or model 5025; PerkinElmer) at two exposures using a 10× air objective or 40× water objective. Analyses of the images were accomplished within the Opera or Columbus environment using standard Acapella scripts.

### Assay Validation and Data Normalization

The statistical reliability of the RVFV MP12 infection was evaluated by calculating for the Z′-factor using the formula: 1−[(3σ_p_+3σ_n_)/|μ_p_−μ_n_|)], where μ_p_, σ_p_, and μ_n_, σ_n_ are the mean (μ) and standard deviations (σ) of both positive (p) and negative (n) controls [20]. The mock-infected and RVFV-infected HeLa cells were used as negative and positive controls, respectively. Percentages of infected cells were normalized with mock-infected and RVFV-infected cells which were considered as 100%, and the values obtained were subtracted from 100 to determine the percent infection inhibition. The same formula was used for all percent infection inhibition calculations unless specified differently. For primary screening, Z′-factor ≥0.5 was used to validate the results of the assay. The hit (i.e., a compound that demonstrates inhibition of virus infection) selection criteria for the primary screening was set at ≥50% inhibition of RVFV infection.

### Hit Confirmation by Dose-Response Curve Analysis

Dose response curve analysis was used to confirm hit compound activity against RVFV, VEEV, EBOV, MARV and LASV. Briefly, HeLa cells were seeded at a concentration of 2×10^4^ cells per well in a 96-well plate. After an overnight incubation at 37°C, hit compounds and references compounds, except for IFN-α, were tested in a 10-point dose-response curve (2-fold serial dilution from 200 µM) assay. IFN-α, was used as reference only in RVFV infections. The assay was performed in the same manner as the primary screening assay described above. Cells subjected to IFN-α were incubated with media containing serial dilutions (2-fold from 3000 units/mL) of IFN-α for 16 h (or overnight) prior to the start of virus infection. Two hours post-infection, cells were washed (one time with 1× PBS) and fresh medium was added back. At the end of virus incubation, cells were fixed and subjected to IFA to visualize the corresponding viral antigen expression as described in the secondary screen above. Each concentration of the hit compound was tested in triplicate. Percent infection inhibition was determined normalizing the percentage of infected cells in compound treated cells with mock treated (0.5% DMSO) and virus infected cells. Data generated from the image analysis of the dose response curve assay were plotted and analyzed using the non-linear regression formula: log (inhibitor) vs. response –variable slope (4 parameters) in GraphPad Prism 6. The EC_50_, defined as the effective concentration resulting in a 50% inhibition of virus infection, was used to evaluate compound activity. Compound toxicity was determined by normalizing cell number of compound treated+virus-infected cells with mock treated (0.5% DMSO)+virus-infected cells, which were considered as 100%. The CC_50_ value, defined as the compound concentration resulting in a 50% reduction in cell viability (based on normalized data) compared with mock infection was used to evaluate cell toxicity. The relative effectiveness of the compound is defined in terms of its selectivity index (SI), a value that indicates the relationship between the compound's effective and toxic concentrations, and is calculated as: SI = CC_50_/EC_50_. It is therefore desirable for a compound to have a high SI value, indicating maximum antiviral activity and minimal cell toxicity.

### Time-of-Addition (ToA) Assay

HeLa cells at a concentration of 2×10^4^ cells per well in a 96-well plate were either mock-infected or treated with compound 2 h prior to infection (pre), concurrent with virus infection (0 h), or at various time points post-infection as indicated in the experiment. Cells were infected with RVFV strain MP12 at an MOI of 10 for 1 h at 4°C. After the 1 h incubation, cells were washed one time with 1× PBS to remove unattached virus and incubated at 37°C with complete media for the duration of the experiment. After 13 h, cells were fixed in 10% Formalin and immunostained to detect viral G protein expression. Experiments were performed in triplicate and the average (± standard deviation) of three independent experiments is shown.

### Quantitative RT-PCR

RVFV RNA yields were determined by Quantitative RT-PCR (qRT-PCR) using TaqMan-based probe sequences as previously described [24]. In brief, total RNA from supernatants or cells were prepared using a MagMax 96 RNA extraction kit (Life Technologies) or RNeasy Plus minikit (Qiagen) respectively following manufacturer's instructions. Purified RNA was then measured with a Quant-IT RiboGreen RNA assay kit (Life Technologies) of which fifty nanograms of RNA was used in the assays. qRT-PCR assays were then performed on an ABI Prism 7900HT sequence detection system with an RNA UltraSense one-step kit (Life Technologies) and TaqMan probes (Applied Biosystems) according to the manufacturers' instructions. Serial 10-fold dilutions of the assayed virus (10^2^ to 10^7^ copies) were used as standards. The viral RNA levels from cell extracts was normalized with the PPIB housekeeping gene. Four wells of a 96 well plate were used to generate one data point and the experiment was done in triplicates. Relative expression levels were determined using the comparative threshold cycle (*C_T_*) method [25].

### Production of Stable Cell Line Expressing RVFV Gn-Gc Polyprotein

A HeLa cell line stably expressing RVFV Gn-Gc polyprotein, referred as HeLa-G was made by transfecting HeLa cells with the expression vector pCAGGS containing the open reading frame of RVFV Gn and Gc genes (kindly provided by Dr. Shinji Makino [26]) and an empty pcDNA3.1 vector expressing a neomycin selection marker at a 8∶1 ratio followed by selection using Geneticin (G418; Life Technologies, USA), an analog of neomycin sulfate, at a concentration of 400 µg/mL. Isolated clones expressing RVFV Gn protein were verified by IFA and western blots analysis.

### High Content-Based Image Analysis to Quantify Changes in Golgi Phenotype and to Evaluate Dose Response Curve Analysis

RVFV-Gn localized to Golgi complex and serves as a Golgi marker. Thus HeLa-G cells were used to quantify changes in Golgi phenotype. HeLa-G cells were seeded at 1×10^4^ the day before the experiment. Cells were mock treated or treated with hit compounds for 12 h, with the exception of IFN-α2a, in which cells were treated overnight prior to start of the experiment. Following treatment, cells were fixed in 10% Formalin, permeabilized with 0.1% Triton-X-100 and immunostained to detect Gn protein expression. Subsequently cells were also stained with Hoechst for nuclear detection. Images were acquired on Opera using 20× air objective, and the images were analyzed with Columbus High Content Imaging software. Individual cells were segmented based on the Hoechst nuclear stain using the “Find Nuclei” building block and using “Select Cell Region” building block, a ring region of 15 pixel width was then created around the individual nuclei that was further masked to classify them as cells. A common boundary was drawn connecting the individual Golgi stacks appearing as spots and spaced less than 2 pixels apart to create a “Golgi mask.” Subsequently, the area of the Golgi mask was evaluated and only those with surface area ≥100 pixels were considered as “intact Golgi.” Percent Golgi was determined by normalizing “Golgi number” per field with the “number of nuclei” in the same field, and the resulting values were then normalized with vehicle control (0.5% DMSO) treated cells. The values obtained were plotted and analyzed using the non-linear regression formula: log (inhibitor) vs. response-variable slope (4 parameters) in GraphPad Prism 6. The GC_50_ was defined as the effective concentration resulting in a 50% reduction in Golgi numbers.

### High-Content Image Analysis of Tubulin

HeLa cells were either mock treated (0.5% DMSO) or treated with BFA (50 ng/mL) for 15 min or nocodazole (33 µM) for 5 h or D011-2120 (50 µM) for 4 h and fixed in 10% Formalin, permeabilized with 0.1% Triton-X-100, and immunostained with tubulin antibody. Subsequently, cells were stained with nuclear Hoechst (33342) dye and Deep red (Cell mask). Images were acquired on an Opera System using a 40× air objective and exported to Columbus High Content Imaging and Analysis Software. Individual cells were segmented based on the Hoechst nuclear stain using the “Find Nuclei” building block and the cell mask deep red stain using “Select Cell Region.” To quantify tubulin phenotype, texture analysis was performed using the Columbus software and the “SER Features” method. The “SER Features” method analyzes the occurrence of typical patters in the intensity structure of an image. The “Ridge Value” was selected as this feature showed the most difference in the intensity pattern of mock-treated vs. nocodazole-treated cells. The ridge values were collected from 4 fields and 3 wells for each condition, and the values were normalized with control treated cells, which were considered as 100%.

## Results

### High Content Image-Based Assay Development and Validation

**Figure 1:**
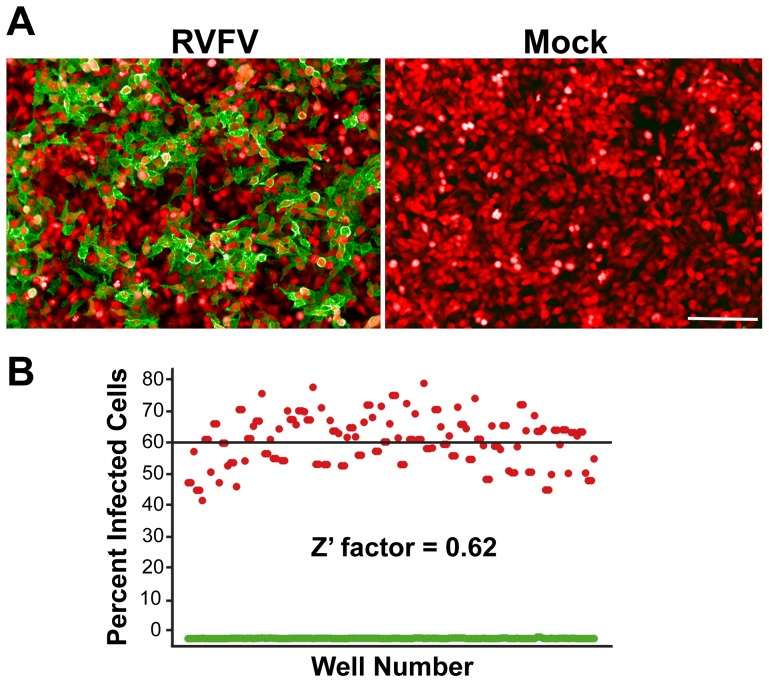
Assay development and validation of the HCI-based RVFV assay. (**A**) IFA of RVFV Gn expression (green) in RVFV-infected or mock-infected HeLa cells (cytoplasm is stained red). (**B**) Scatter plot distribution of percentage of cells expressing RVFV Gn acquired from mock-infected (green) or RVFV-infected (red) HeLa cells and the evaluation of degree of separation between the two controls (Z′-factor). Data are derived from triplicate experiments performed on three separate days. Horizontal line represents the average percent value of RVFV-infected cells.

HeLa cells were considered the optimal cell line to use for assay development as the cells are permissive to RVFV infection, are the optimal size for image analysis, possess a functional interferon response, and they are relatively easy to grow. Selection criteria for the appropriate cell seeding density included having a sufficiently high number of cells but with enough spatial distribution for proper identification during image acquisition. After testing various seeding densities of HeLa cells, the seeding density of 2×10^4^ cells per well was selected as minimal variations among replicates were noted without compromising proper segmentation of cells (data not shown). RVFV infection in HeLa cells was visualized by IFA detection of the RVFV Gn protein using the 4D4 mAb and confocal imaging using the Evotec Technologies High-Throughput Cell Analyzer Opera (Fig. 1A). Expression of this protein on the cell surface is indicative of virus egress from the infected cell. For the RVFV infection, a MOI of 1.0 and incubation time of 24 h was used since it achieved an infection rate of ∼60% and allowed multiple rounds of virus replication that facilitated the screening of active compounds that target different stages of virus life cycle. Finally, the RVFV infected cells were classified as those cells having G signal within the defined boundaries of a cell and greater than a pre-defined threshold level. Several additional parameters (∼50; data not shown) were acquired from the images, some of which include the nuclear and cytoplasmic intensities of the G signal, nuclear size and nuclear intensities. However, for the screening purposes described in this section, only cell number and the infected cell number was used to determine the percentage of infected cells.

The Z′-factor was evaluated to test the robustness of the assay in the 96-well plate format. Figure 1B shows a representation of the validation process done for the RVFV infection in HeLa cells. Cells were dispensed in a 96-well plate and half (48 wells) were mock-infected (0.5% DMSO only) while the other half were infected with RVFV. Mock-infected wells were considered as negative controls, while RVFV-infected wells as positive controls. After the viral infection period and IFA, image analysis of the acquired images revealed the infection percentage, cell number based on cell nuclei detected, and other pre-defined parameters. The Z′-factor was calculated using average and standard deviations of the percent infection of the positive and negative controls as described in methods section [27]. The experiment was performed in triplicate on three separate days, and the calculated Z′-factor was 0.62. A Z′-factor ≥0.5 indicates a statistically reliable separation between positive and negative controls.

### Screening of the ChemDiv Protease Inhibitor Library

**Figure 2:**
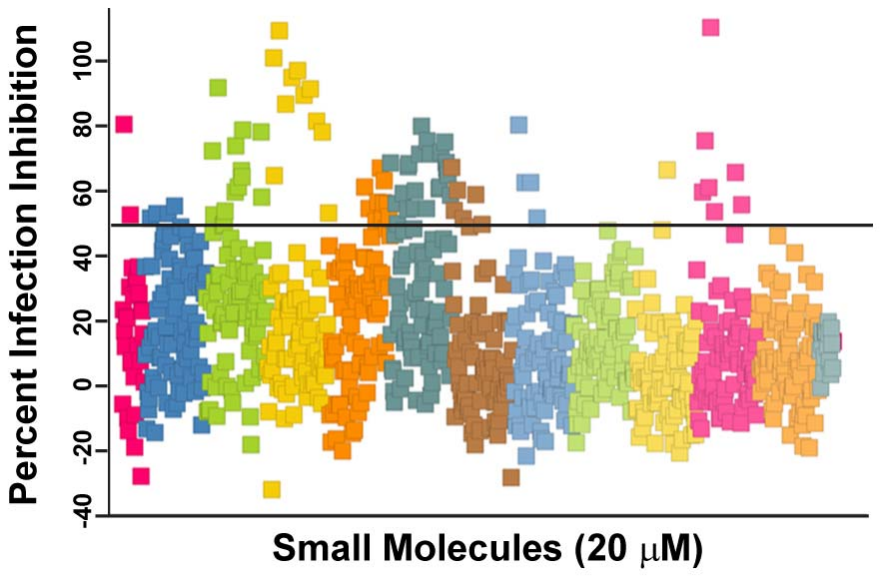
Primary screening of protease inhibitor library. Scatter plot of primary screening data where the percentage of infected cells was normalized with mock-treated, RVFV-infected cells to determine the percentage of infection inhibition. A collection of 849 compounds from the ChemDiv protease inhibitor library in 13 96-well plates were screened at a 20 µM concentration against RVFV MP12 strain in HeLa cells. Data from each of the 13 plates are represented by same color. Hit compounds having greater than 50% antiviral activity (represented by horizontal black line) were selected for secondary screening.

A total of 849 compounds from ChemDiv protease inhibitor library were used for antiviral screening. The library was screened at 20 µM concentration against RVFV and primary hits were selected based on the criteria: ≥50% inhibition and ≥50% cell number compared to positive controls. Figure 2 shows a scatter plot of the primary screening data where percent infected cells were normalized with mock-treated RVFV-infected cells to determine the percent infection inhibition (see methods). Thirteen separate master compound plates were used and are represented as separate colors in Figure 2. Many of the hit compounds clustered together on the scatter plot since the compounds were grouped together on the plate by primary chemical structure. Sixty-one compounds were initially selected as primary hits (exhibiting ≥50% inhibition), although we suspected some of these may be due to edge well effects. Upon repeat screening, the compounds with suspected edge well effects proved not to be active and were eliminated from subsequent analysis. Thus, a total of 34 compounds (4.0%) exhibited ≥50% inhibition against RVFV (shown above the black horizontal line at 50% infection inhibition in Figure 2).

**Figure 3:**
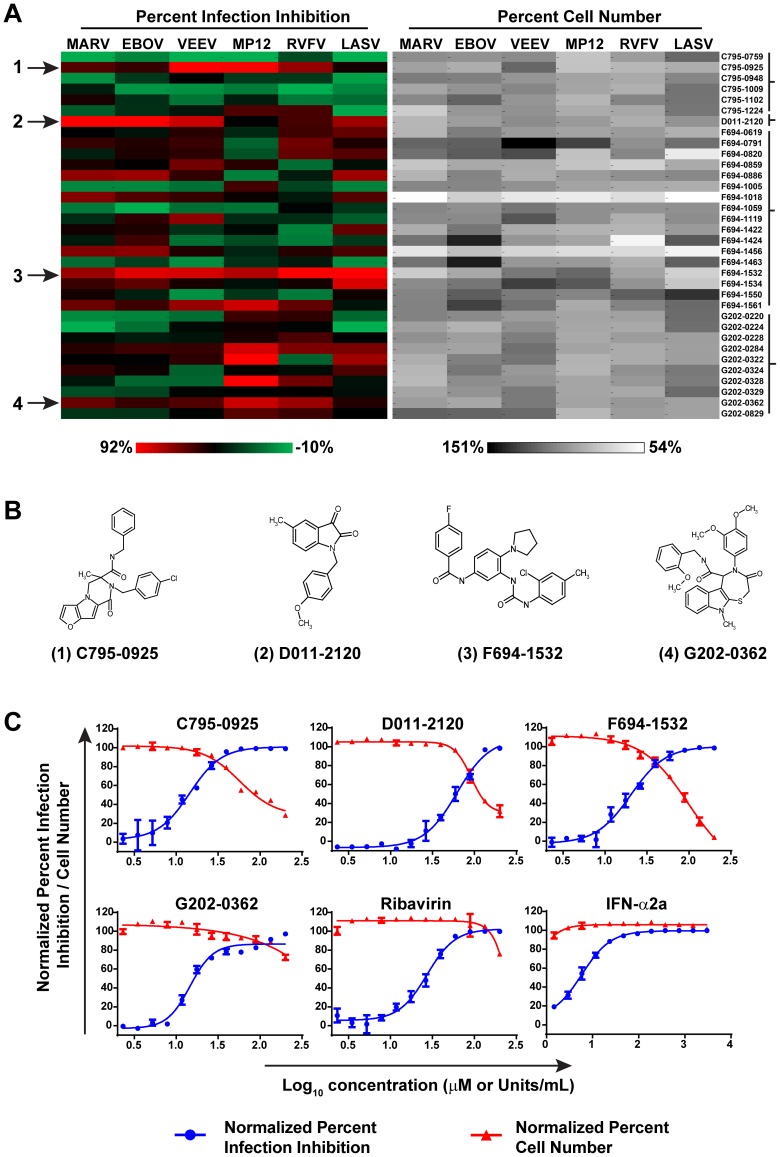
Secondary screening of hit compounds against multiple RNA viruses and validation determined by dose response curve analysis of lead compounds and reference compounds. (**A**) Heat map showing relative percent infection inhibition (left panel) and percent cell number (right panel) of compounds (10 µM concentrations) against multiple RNA viruses. The four hit compounds are marked with arrows (**A**) and their primary structures are shown (**B**). (**C**) Dose response curve analysis of the four lead compounds and the reference compounds ribavirin and IFN-α2a. Percent infection inhibition is shown in blue and the percent cell number is shown in red.

In order to determine if the primary hits had broad-spectrum activity against other highly pathogenic RNA viruses, they were further screened against multiple viruses, including EBOV, MARV, VEEV, LASV, and the virulent RVFV ZH501 strain, in addition to MP12. Figure 3A shows the generated heat map of percent virus infection inhibition and percent cell viability. As can be seen in the heat map, most of the compounds had varying degrees of activity across the multiple RNA viruses tested. The 34 primary hit compounds were classified into four distinct groups based on their primary chemical structure as shown in brackets in Figure 3A. Four compounds, each comprising a unique primary chemical structure (Fig. 3B), were selected based on activity against three or more of the viruses tested (Fig. 3A). All other primary hit compounds were chemical analogs of these four compounds. These compounds were selected for further study to determine their EC_50_, CC_50_, SI, and mechanism of action against RVFV.

Antiviral activities of the four hit compounds against RVFV were determined by dose response curve analysis in HeLa cells. The EC_50_, CC_50_ and SI were determined for the four compounds and two reference compounds, ribavirin and IFN-α2a, known inhibitors of RVFV. Figure 3C shows the dose response curve analysis of the four hit compounds and two reference compounds, with percent infection normalized against RVFV-infected HeLa cells. All compounds tested showed inhibition of RVFV-infected HeLa cells in a dose-dependent manner (Fig. 3C). Furthermore, all compounds tested had a SI of greater than 1, indicating that the virus inhibitory effects were apparent before the cytotoxic effects appeared. G202-0362 had the lowest effective dose (EC_50_ = 13.6 µM), and significantly, appeared to have no toxic effects on the HeLa cells at the concentration used in the experiment (Table 1). C795-0925 had a similar effective dose (EC_50_ = 14.4 µM), albeit the compound was more toxic to cells (CC_50_ = 56.7 µM) compared to G202-0362. D011-2120 had the highest effective dose (EC_50_ = 61.5 µM) and consequently the lowest SI (1.5) (Table 1). The effective doses of ribavirin and IFN-α inhibited RVFV were consistent with what has been reported in the literature [17], [28], [29]. In order to examine the potential that the inhibitory effects of these compounds were due to an artifact of using a cancer cell line (HeLa), we repeated the screens of these four compounds and references compounds against RVFV-infected human small airway epithelial (SAEC) cells, a primary cell line derived normal human lung tissue. The effective doses of all the compounds, except for IFN-α2a, were similar to those observed in HeLa cells (Table 1). IFN-α was approximately 10-fold less effective against RVFV infection in SAEC compared to in HeLa cells. IFN-α has several targets in mammalian cells and therefore, although highly effective, is not an ideal model to compare with other drugs. Interestingly for C795-0925, while the EC_50_ was comparable between the two cell lines, the compound was less toxic to SAEC (CC_50_ = 110.6 µM).

**Table 1:** Antiviral activity of the four hit compounds and reference compounds against RVFV-infected HeLa cells or SAEC primary cells.

	HeLa cells			SAEC primary cells		
Compound	EC_50_ (µM)	CC_50_ (µM)	SI	EC_50_ (µM)	CC_50_ (µM)	SI
C795-0925	14.4	56.7	3.9	12.3	110.6	9.0
D011-2120	61.5	91.4	1.5	62.4	98.0	1.6
F694-1532	20.0	98.3	4.9	23.5	51.3	2.2
G202-0362	13.6	ND	>16	11.9	ND	>16
Ribavirin	27.2	ND	>16	29.5	ND	>16
IFN-α2b	5.9 U/mL	ND	>16	33.5	ND	>16

HeLa cells and SAEC were infected at MOIs of 1.0 and 0.5, respectively for 24 h.

SI is determined by the formula: CC_50_/EC_50_.

ND, not determined.

The hit compounds' potency against other pathogenic viruses including VEEV, EBOV, MARV and LASV were further evaluated by does response analysis (Table 2) and compared to ribavirin's antiviral activity. In VEEV infections, all 4 compounds were more potent than ribavirin (a broad spectrum antiviral agent) while C795-0925 was most potent among all with EC_50_ = 10.2 µM. G202-0362 was most potent in filoviruses EBOV and MARV with EC_50_ = 16 µM and 34 µM respectively and more potent than ribavirin. However, in LASV infections, ribavirin was most effective compared to all 4 compounds. Collectively, these data show that these compounds were effective against more than one virus.

**Table 2:** Antiviral activity of the four hit compounds and reference compounds against VEEV, EBOV, MARV and LASV infected HeLa cells.

Viruses	Compound	EC50 (µM)	CC50 (µM)	SI
VEEV	C795-0925	10.2	63.7	6.2
VEEV	D011-2120	41.6	42	1.0
VEEV	F694-1532	15.2	23.8	1.6
VEEV	G202-0362	48.9	ND	>4.1
VEEV	ribavirin	75	ND	>2.7
EBOV	C795-0925	46.6	ND	>4.3
EBOV	D011-2120	54.1	65.6	1.2
EBOV	F694-1532	117.6	ND	>1.7
EBOV	G202-0362	16.1	ND	>12.5
EBOV	ribavirin	60.1	ND	>3.3
MARV	C795-0925	57.4	ND	>3.5
MARV	D011-2120	87.1	90.6	1.0
MARV	F694-1532	143.3	167	1.2
MARV	G202-0362	34.1	ND	>5.9
MARV	ribavirin	82.2	ND	>2.4
LASV	C795-0925	81.4	103.2	1.3
LASV	D011-2120	46.8	47.2	1.0
LASV	F694-1532	80.4	ND	>2.5
LASV	G202-0362	ND	ND	ND
LASV	ribavirin	6.8	ND	>29.4

HeLa cells were infected with VEEV (MOI = 0.5 for 20 h), EBOV (MOI = 3 for 48 h), MARV (MOI = 3 for 48 h) or LASV (MOI = 1 for 24 h).

SI is determined by the formula: CC_50_/EC_50_.

ND, not determined.

### Hit Compounds Target Viral Replication and/or Viral Egress, but Not Entry

**Figure 4:**
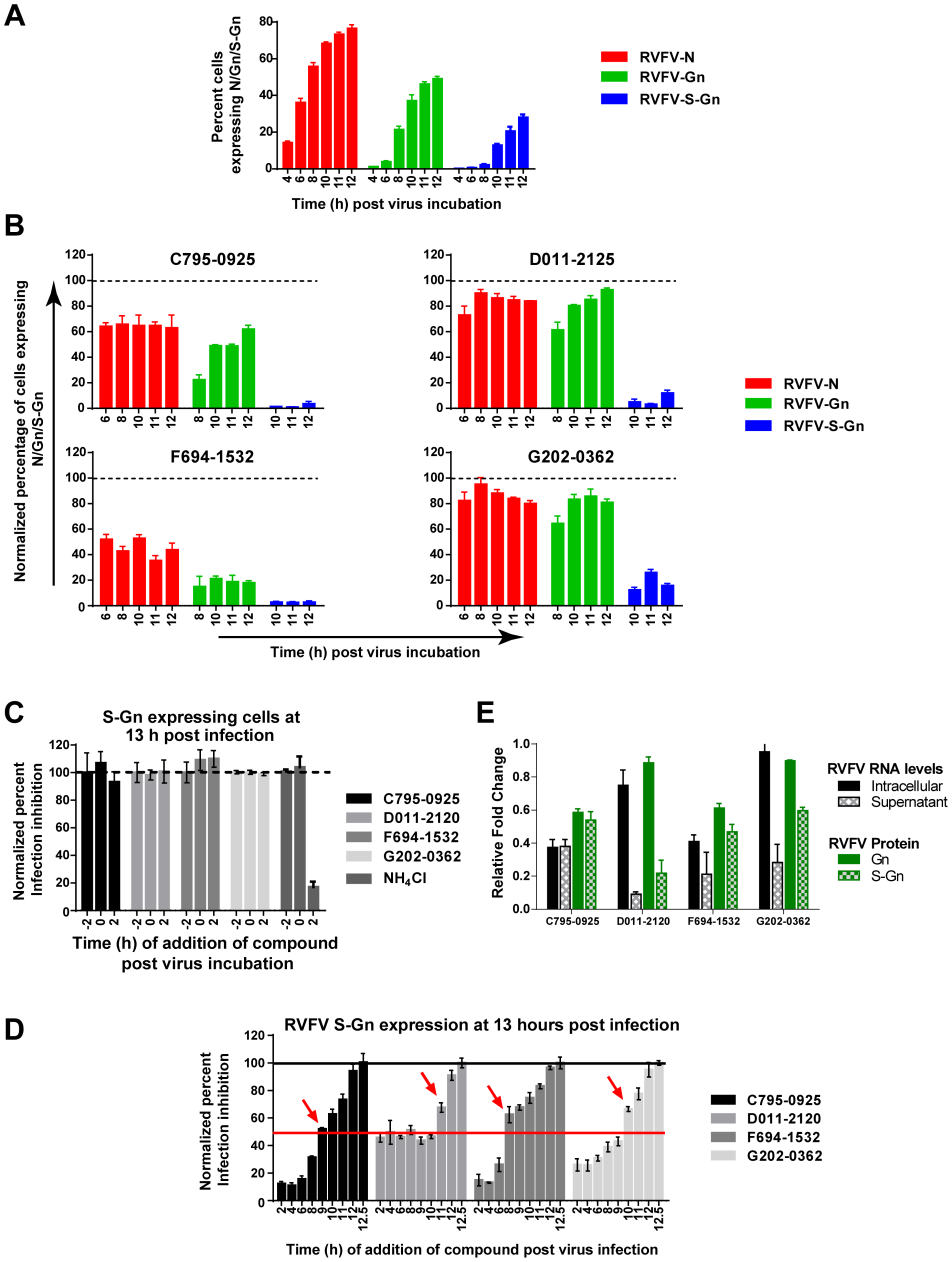
Hit compounds target virus replication and/or virus egress, but not virus entry. (**A**) Kinetics of N, Gn or S-Gn expression in RVFV-infected HeLa cells (MOI = 10) during a single replication cycle. Cells were fixed at various time points post-infection (PI) (0 h) as indicated in the x-axis. (**B**) Kinetics of N, Gn or S-Gn expression in RVFV-infected HeLa cells with hit compound treatment. (**C**) Time-of-addition assay in which HeLa cells were either mock treated or treated with the hit compounds at 2 h before prior to (−2), at the same time (0), or 2 h after virus infection. Cells were fixed at 13 h PI and immunostained to detect S-Gn expression. As a control for viral cell entry, cells were treated with 20 µM NH_4_Cl, a known inhibitor of endocytosis. (**D**) Time-of-addition assay in which HeLa cells were either mock treated or treated with the hit compounds at various times PI as indicated on the x-axis. Cells were fixed at 13 h PI and immunostained to detect S-Gn expression. (**E**) Relative changes in the RNA levels at cellular level or in the supernatants of compound treated cells at 12 h PI. Cells were mock treated or treated with the 4 hit compounds from 2 h prior to the start of infection through the entire duration of infection. In a duplicate experiment the percentage of cells expressing Gn and S-Gn was determined by HCI of the immunostained cells. The relative fold change was determined by normalizing data of compound treated cells with values of corresponding mock treated cells.

RVFV infection follows an ordered sequence of steps that can be visualized using IFA for the early nucleocapsid (N) and late glycoprotein (Gn) expression in infected cells [28]. Compounds targeting any stage of the viral life cycle should alter either the kinetics or localization of viral protein expression. Thus we used time-of-addition (ToA) assays, adding compounds at various time points before or after virus infection, combined with HCI analysis to determine at which stage of the viral life cycle the compound is acting. To examine the kinetics of N, Gn, and cell surface exposed Gn (referred to as S-Gn) expression, HeLa cells were infected with RVFV MP12 virus (time = 0 h), and the percent of cells expressing N, Gn, and S-Gn were determined by IFA at 4, 6, 8, 10, and 12 h post-infection (PI). N protein expression was apparent at 4 h PI and steadily increased throughout the 12 h infection cycle, at which time approximately 70% of the cells expressed the protein (Fig. 4A). Gn expression was first apparent at 8 h PI and steadily increased throughout the infection cycle to a peak of approximately 40% of the cells expression the protein at 12 h PI. Cell surface exposed Gn (S-Gn) protein expression is indicative of virus egress. Consistent with this, S-Gn was first apparent at 10 h PI and peaked at 12 h PI in approximately 25% of the cells (Fig. 4A) and corresponds to one round of replication of the RVFV life cycle [28]. Release of virus into the extracellular medium was further confirmed by quantitative PCR analysis (Q-PCR) and plaque assay (data not shown). Compounds targeting virus entry or virus replication will show aberrant kinetics in the cytoplasmic expression of N and Gn. Alternatively, if the compound only targets virus egress then changes in the kinetics of S-Gn expression, and not cytoplasmic Gn or N expression, should be observed. Therefore, the kinetics of viral N, Gn, and S-Gn expression in the presence of compound was examined. The percentage of cells expressing N, Gn or S-Gn upon compound treatment was normalized with mock treated and infected cells, which was considered as 100% (Fig. 4B). Treatment with all compounds significantly reduced the expression of S-Gn by 70–100%, indicative reduction of infectious virus getting to the cell surface. However, treatment with F694-1532 and C795-0925 also significantly reduced expression of N and Gn, suggesting these compounds exhibited their effects earlier in the virus life cycle. While treatment with D011-2120 and G202-0362 reduced S-Gn expression approximately 90%, they had only minor effect (∼10% reduction) on N and Gn expression (Fig. 4B). These data suggest that D011-2120 and G202-0362 are targeting virus egress, while F695-1532 and C795-0925 may be targeting steps earlier in the virus life cycle.

To further define the stage of the viral life cycle the compounds are acting, compounds were added either 2 h prior to infection (−2 h), at the time of infection (0 h), or 2 h post-infection (2 h). Acid conditions (pH<5.0) inside the endocytosed virus-containing vesicles are necessary to promote virus-host membrane fusion [12], [28]. Thus, we used NH_4_Cl (20 mM) as a control as it targets the last stage of the virus entry pathway by neutralizing the pH inside the acidic compartments within the cell. Figure 4C shows that NH_4_Cl inhibited RVFV infection when cells were treated at early time points (−2 h or 0 h), but not at the later time point (2 h), consistent with its role as an entry inhibitor. Conversely, levels of virus inhibition with each of the four hit compounds added 2 h PI were similar to levels of inhibition when the compounds were added at −2 h or 0 h. (Fig. 4C). These data suggest that the observed inhibitory effects of the four hit compounds were not due to a block in the virus entry pathway.

In order to examine how the compounds affect subsequent stages of the virus life cycle, compounds were added to virus-infected HeLa cells at 2, 4, 6, 8, 9, 10, 11, 12, or 12.5 h PI and the percentage of infected cells were evaluated at 13 h PI using IFA for S-Gn expression, which is indicative of virus egress.

More than 50% of infection was rescued when F694-1532 or C795-0925 were added at 8 h or 9 h PI respectively (Fig. 4D, marked with red arrows), which suggested that compounds mostly targeted viral RNA replication or viral protein expression. Not surprisingly, D011-2120 and G202-0362, which appeared to target virus egress, did not recover restriction until late stages (>10 h for 50% infection recovery). Collectively these data suggested that C795-0925 and F694-1532 targeted viral RNA transcription or protein synthesis and not virus entry or egress, while D011-2120 and G202-0362 targeted egress. To further confirm these observations, viral RNA levels were evaluated to determine compound mediated effects on RVFV life cycle as described below.

The compound mediated effect on virus replication was determined by measuring the amount of viral RNA that accumulated inside infected cells or in their supernatants at the end of one life cycle. The viral RNA in the supernatants is a measure of virus egress. Cells were mock- treated or incubated with any of the four hit compounds beginning 2 hours prior to the start of infection. Twelve hours post-infection, the cell extracts or supernatants were collected separately, and the viral RNA levels were evaluated by qRT-PCR. The RNA levels in compound-treated cells were normalized with mock-treated cells to calculate the relative change in RNA levels (Fig. 4E). Alternatively, in the duplicate experiment, cells were subjected to IFA of Gn and S-Gn protein expression. HCI was then applied to evaluate the percentage of cells expressing Gn or S-Gn, and the relative change was calculated. (Fig. 4E). As expected, D011-2120 and G202-0362 treatments that targeted virus egress resulted in low levels of 9% and 28%, respectively of viral RNA accumulation in the supernatants as compared to 75% and 95%, respectively inside the cells. In contrast, viral RNA levels inside the cells and in the supernatants were similar for cells treated with C795-025 and F694-1532. In addition, these data corroborated with the viral protein expression levels inside cells (Gn) or on the surface of cells (S-Gn). Collectively, these data suggest that C795-0925 and F694-1532 targeted viral RNA transcription or protein synthesis, and not virus entry or egress, while D011-2120 and G202-0362 targeted egress. HCI was further applied to understand the mode of action as described below.

### D011-2120 Inhibits RVFV Glycoprotein Gn Localization by Disrupting Golgi Complex

**Figure 5:**
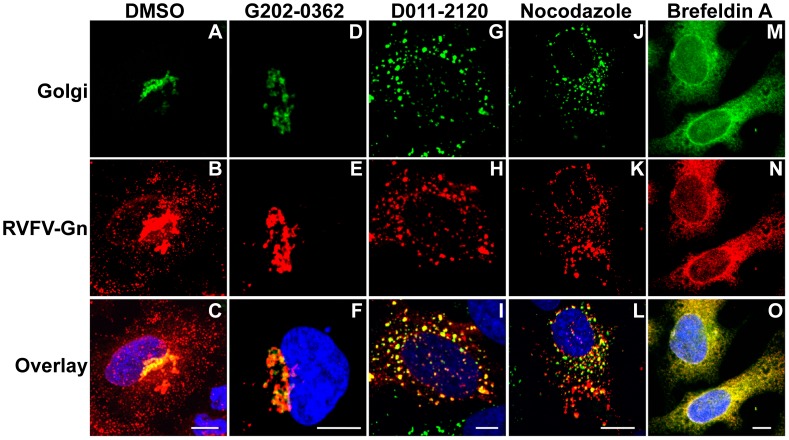
D011-2120, but not G202-0362 inhibits RVFV Gn trafficking by disrupting the Golgi complex. HeLa cells were transduced to express a GFP-fused Golgi marker and were treated with (**A–C**) vehicle control (0.5% DMSO), (**D–F**) G202-0362 (20 µM), (**G–I**) D011-2120 (50 µM), (**J–L**) nocodazole (33 µM) at 2 h PI or (**M–O**) Brefeldin A(100 ng/mL) at 20 min before stopping the infection with RVFV MP12 strain (MOI = 10). Cells were fixed at 10 h PI and immuostained to detect RVFV Gn localization (Red) and stained with Hoechst 33342 to visualize nuclei (blue).

RVFV envelope glycoproteins localize to the Golgi complex and mediate virion assembly and budding into the Golgi lumen [13], [14], [30]. Subsequently, based on similarities with other bunyaviruses, it is believed that virus egress occurs by the fusion of the virion-containing Golgi vesicles with the cellular plasma membrane. Because D011-2120 and G202-0362 appeared to target virus egress, HCA was applied to determine if the virus trafficking from the Golgi complex to plasma membrane was affected. Golgi complex was visualized by transducing cells overnight with baculovirus expressing fusion construct of a Golgi marker and green florescent protein (GFP). RVFV G expression in cytoplasm is detectable by 10 h PI (Fig. 4A) and therefore this time point was used to interrogate any potential compound-mediated changes in G localization within the Golgi complex of infected cells. At 10 h PI, cells treated with 0.5% DMSO alone displayed a bright G signal localized to one side of the nucleus, which is consistent with the location of the Golgi complex (Fig. 5A–B). A portion of the G protein co-localized with Golgi as evidenced by the bright yellow staining when the images were overlaid (Fig. 5C). Furthermore, numerous G stained spots (Fig. 5B–C), presumably virus containing Golgi vesicles were found in the cytoplasm (marked with white arrow). G202-0362 treated cells, similar to DMSO treated cells, expressed G that co-localized with Golgi apparatus (Fig. 5D–F). However the numerous G stained spots in the cytoplasm that were observed in DMSO treated cells were not observed. Therefore it is possible that G202-0362 is targeting virus assembly, virus budding into Golgi lumen or the subsequent release of the virus containing Golgi vesicle into the cytoplasm.

On the contrary, D011-2120 treated virus-infected cells resulted in Golgi dispersion (Fig. 5G–I). G remained localized with the disrupted Golgi. The effects of compounds on Golgi and G protein localization were compared to that of two well-known Golgi-disrupting compounds, nocodazole and brefeldin A (BFA). Nocodazole is a microtubule-disrupting agent and induces a slow dispersal of the juxtanuclear Golgi to peripheral sites [31] (Fig. 5J–L), while BFA causes a rapid disassembly and redistribution of the Golgi into the endoplasmic reticulum (ER) [32], (Fig. 5M–O). D011-2120 and nocodazole had same effect on Golgi dispersion (compare Fig. 5I and 5L) suggesting a common mechanism of action. These results suggest that D011-2120, exerts its antiviral activity by disrupting the Golgi complex in a manner similar to nocodazole, but distinct from BFA.

### High-Content Image-Based Analysis of Changes in Golgi Phenotype by the Hit Compounds

**Figure 6:**
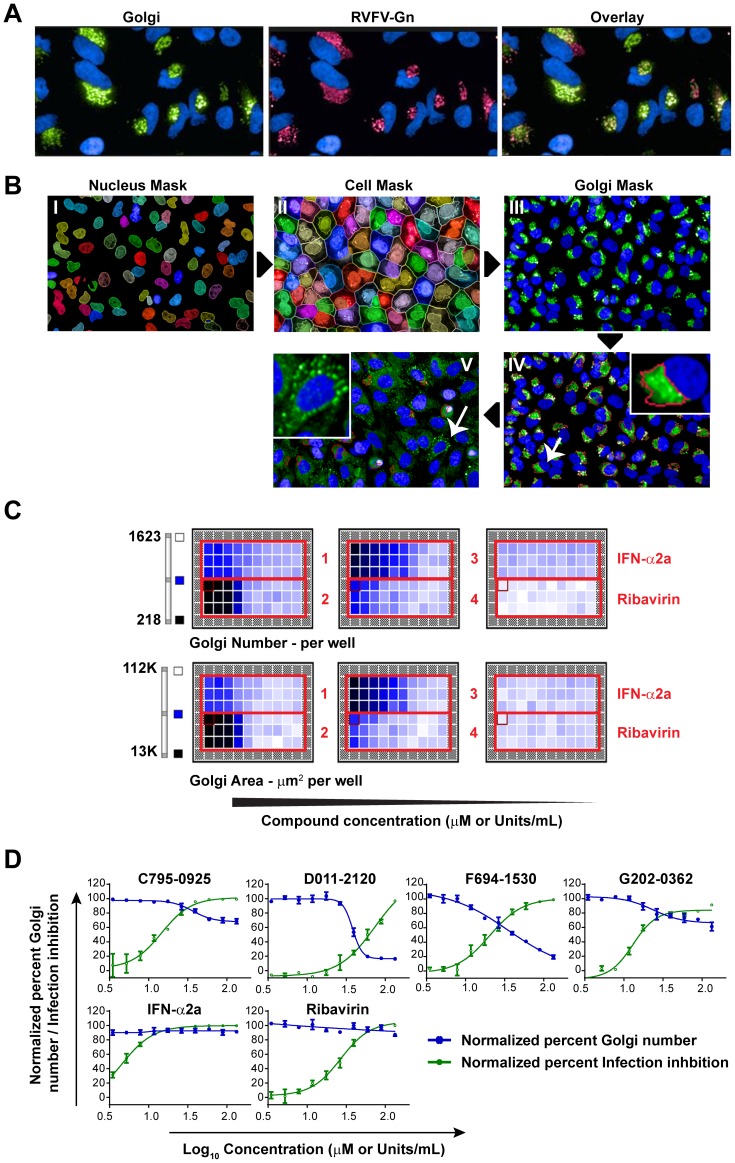
Development of a HCI-based assay to assess phenotypic changes to the Golgi. (**A**) IFA showing co-localization of RVFV Gn (Red) and a GFP-fused Golgi marker (Green) in a HeLa-G cell line that stably expressed RVFV Gn protein. (**B**) Software development for HCI-based analysis of Golgi. Acquired confocal images of nucleus (I), cell (II) and the Golgi complex (III) in mock-treated HeLa cells. The juxtanuclear Golgi is qualified as “intact Golgi” by image analysis and is marked by the red boundary around the clustered Golgi stacks (insert, IV) in mock treated cells. In contrast, image analysis disqualified many of the numerous scattered spots in the cytoplasm of D011-2120-treated cells as “intact Golgi” (insert, V). (**C**) Heat map of Golgi number and Golgi area by HCI-based analysis with increasing concentrations of the hit compounds and reference compounds. (**D**) Dose response curve analysis of lead compounds showing an increased reduction in Golgi as a function of compound concentration. Percent Golgi number is shown in blue and percent infection inhibition is shown in green.

We further developed HCI analysis to quantify alterations in Golgi structures, to estimate GC_50_ values (effective concentration of compound that disrupts 50% of the Golgi complexes) of D011-2120 targeting the Golgi, and to screen other compounds that may also target the Golgi. HCI-based analysis was developed to quantify changes in Golgi phenotype. For this, a HeLa cell line that stably expresses RVFV Gn was generated (HeLa-G). In the absence of viral RNP complex, Gn remains in the Golgi ([14] and reviewed in [33]) and therefore IFA of Gn served as a Golgi marker. IFA showed specificity in the localization of RVFV Gn with Golgi marker (Fig. 6A). Images of Gn stained HeLa-G that were mock-treated or treated with D011-2120 were used further for the development of HCI-based assay for cellular Golgi distribution. Figure 6B shows the individual steps that allowed successful segmentation of nuclei (I), cells (II) and the Golgi complex (III). The image analysis was applied such that the intact juxtanuclear Golgi appeared as one “intact Golgi” (see methods). Figure 6B–IV (see insert) shows a red boundary generated by image analysis to classify “intact Golgi.” D011-2120 treated cells showed Golgi staining as numerous spots dispersed throughout the cytoplasm, which was disqualified as ‘Intact Golgi” by image analysis program (Fig. 6B–V, see insert). This image analysis was further applied to determine how the hit compounds modulated Golgi phenotype in HeLa-G cells that were treated with increasing concentrations of the hit compounds or the reference compounds Ribavirin or IFN-α (Fig. 6C). Several features of the Golgi complex were generated, including “Golgi number” and “Golgi area” per well, and a heat map thus generated shows a clear reduction in Golgi “number” or “area” in wells with higher concentration of D011-2120. The “Golgi number” parameter was further used to determine the GC_50_ values by dose response curve analysis (Fig. 6D), which were compared to the compounds' EC_50_ as shown in Table 3. The GC_50_ value for D011-2120 was less than half that of its EC_50_ value. The ratio of GC_50_ to EC_50_ was further calculated and was determined to be 0.62. A ratio of less than 1 suggests that the compound's antiviral activity is related to its ability to disrupt the Golgi complex. Other compounds had lower GC_50_ values, but when compared to their EC_50_, none of their GC50/EC50 ratios were below 1 (Table 3). Surprisingly, F694-1532 treated cells also resulted in a decrease in Golgi numbers. However, further analysis showed that this compound inhibited expression of Gn or any other exogenously introduced gene such as GFP, but did not disrupt Golgi phenotype (data not shown).

**Table 3:** Golgi disruption properties of the four hit compounds and reference compounds derived from HCI-based analysis of HeLa-G cells.

Compound	EC_50_ (µM)	GC_50_ (µM)	GC_50_/EC_50_	%GN_L_
C795-0925	14.4	36.9	2.6	63.4
D011-2120	61.5	38.2	0.6	16.4
F694-1532	20.0	35.3	1.8	19.5
G202-0362	13.6	23.7	1.7	61.2
Ribavirin	27.2	ND	>16	87.2
IFN-α2b	5.9 U/mL	9.9 U/mL	1.7	91.2

GC_50_, effective concentration of compound that disrupts 50% of the Golgi complexes.

% GN_L_, which is the lowest % of Golgi number achievable at the compound's highest concentration.

ND, not determined.

### D011-2120 Acts by Depolymerizing Microtubules Similar to Nocodazole

**Figure 7:**
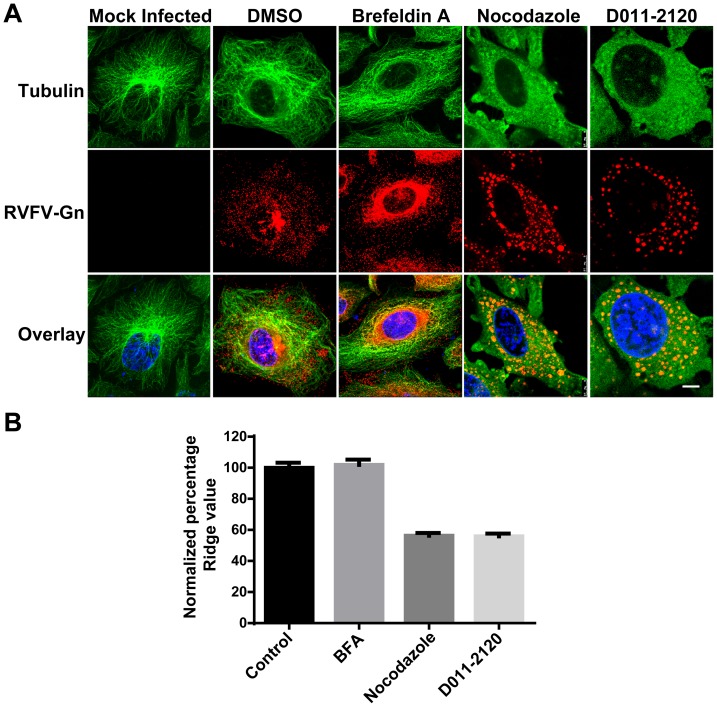
D011-2120 depolymerizes microtubules similar to nocodazole. (**A**) IFA of HeLa cells that were mock infected of infected with MP12 virus for 12 h and either were mock treated or treated with vehicle control (0.5% DMSO), Brefeldin A (100 ng/ml) nocodazole (33 µM), and D011-2120 (50 µM) for 15 min, 4 h or 10 h respectively prior to stopping infection. Cells were immunostained to detect tubulin (green) or RVFV Gn (red). (**B**) HeLa cells in a 96 well plate were treated with D011-2120 or reference compounds for 4 h and immunostained with tubulin antibody. Acquired images were subjected to pattern and ridge analysis (Columbus software) to evaluate differences in microtubulin organization within the cells. Each data point is derived from eight replicates. Percent ridge values were obtained by normalizing each data point with 0.5% DMSO treated cells, which was considered 100%.

Because of the similarities observed in Golgi staining patterns in cells treated with D011-2120 and cells treated with nocodazole, we hypothesized that they are both acting by a similar biochemical mechanism. It is well-known that nocodazole exerts its effects by inhibiting polymerization of microtubules [34], which are made up of linear polymers of tubulin that undergo continual assembly and disassembly to form a dynamic filamentous microtubule network within cells. Thus, we reasoned that D011-2120 may also be acting to inhibit this process. To test this, the kinetics of Golgi complex dispersion in HeLa cells exposed to either vehicle control (0.5% DMSO), D011-2120, nocodazole, or BFA were examined by live cell imaging. Our previous data indicated that a 4 h exposure to both D011-2120 and nocodazole was optimal for cells to exhibit the Golgi dispersion phenotype (data not shown). Thus, HeLa cells infected with MP12 virus for one life cycle (12 h) and treated with D011-2120 or Nocodazole during the last 4 h of infection or with BFA during the last 15 min of infection. Tubulin was visualized by IFA. As expected, treatment with either the vehicle control (0.5% DMSO) or BFA showed predominantly filamentous microtubule network (Fig. 7A). In contrast, cells treated with either nocodazole or D011-2120 showed diffused pattern of tubulin staining (Fig. 7A). To further quantify tubulin phenotypic changes, HeLa cells in a 96-well plate were treated with D011-2120 or reference compounds for 4 h and immunostained with tubulin antibody. The images were further subjected to HCA to quantify the changes in the pattern of microtubule organization using the “Ridge Value” of the patternanalysis program (Columbus Software). The “ridge” feature best distinguished the filamentous pattern in control cells from the diffused staining of tubulin in the compound treated cells (Fig. 7B). The ridge value for cells treated with D011-2120 was similar to cells treated with nocodazole, which were approximately half that of cells treated with BFA or mock-treated (Fig. 7B).

**Figure 8:**
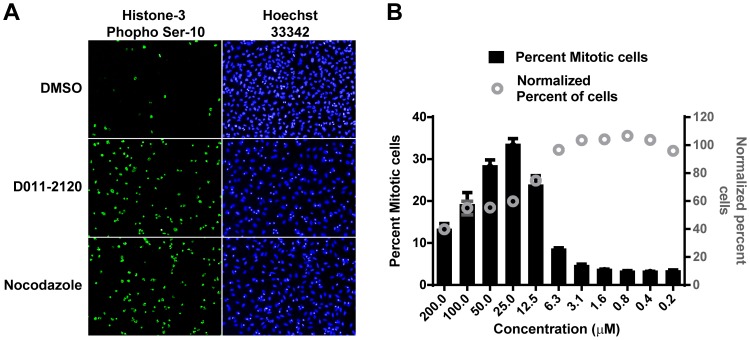
D011-2120 induces mitotic arrest. (**A**) Representative images of HeLa cells treated with vehicle control (0.5% DMSO), D011-2120 (50 µM), or nocodazole (33 µM) for 24 h and immunostained with Histone 3 phospho Serine 10 (pH3-S10) antibody to detect mitotic cells (green). Nuclei are stained with Hoechst 33342 (blue). (**B**) Graph of data acquired using HCI-based analysis showing the percent mitotic cells treated with increasing concentrations of D011-2120 as indicated on the X-axis.

Because the separation of chromosomes requires functional spindle fibers that are made of microtubules, microtubule depolymerizing agents, such as nocodazole, are known to arrest cells in the G2/M phase of the cell cycle [35]. To determine if D011-2120 induces mitotic arrest in treated HeLa cells, cells were treated with various concentrations of the compound and immunostained to detect the expression of phosphorylated histone 3 at serine number 10 (H3-pS10), a known marker of cells undergoing mitosis and that is associated with chromatin condensation [36]. As compared to mock-treated (0.5% DMSO) cells, there was a significant increase in the number of cells expressing H3-pS10 when cells were treated for 24 h with 25 µM of D011-2120 or 3.3 µM nocodazole (Fig. 8A). Furthermore, the effect was dose dependent with the number of cells expressing H3-pS10 increasing with increasing concentration of D011-2120 up to 25 µM (Fig. 8B). Concentrations above this amount were toxic to the cells. Taken together, these data suggest that D011-2120 acts by depolymerizing microtubules resulting in mitotic arrest.

## Discussion

RVFV, along with VEEV, MARV, EBOV and LASV all cause serious disease in humans and animals (RVFV and VEEV). As such, they are classified by NIAID as category A priority pathogens with bioterrorism potential. In addition to being highly virulent, there is currently no FDA approved antivirals to treat any of them. In this study, we applied HCI-based analysis as a screening tool to discover novel compounds with antiviral activity against these highly pathogenic viruses and to elucidate their mode of action. Image-based high-throughput screening of potential antiviral compounds is an extremely effective drug discovery tool as it can not only determine antiviral activity, but can also measure many aspects of the viral life cycle and virus interaction with multiple host cellular components. However, many potential antiviral compounds could be targeting a cellular component that is essential for normal functioning of host cells. It is at this juncture that HCI-based screening has promise over traditional high-throughput screening assays. The sophisticated image analysis in HCS offers more information including intensity of expression, localization and pattern of expression (i.e., texture) of the fluorescent target. Since viral infection follows a specific sequence of viral gene expression, any changes in the kinetics of viral protein expression patterns can provide useful insights into the stage of the virus life cycle targeted by the compound. This information can, therefore, be helpful in forming a hypothesis regarding which specific virus-host protein interaction(s) the compounds are acting on.

To discover novel compounds with broad spectrum antiviral activity, the strain MP12 of RVFV served as a model system for primary screening. This attenuated strain was previously shown to faithfully recapitulate the replication properties of wildtype RVFV [15] and unlike the wildtype virus, can be handled safely under BSL-2 laboratory conditions. Thus, we used MP12 virus-infected cells to set the optimal system parameters, which included cell type, cell number, duration and multiplicity of infection, to achieve a statistically reliable high-throughput screening assay with a Z′-factor of 0.62 (Fig. 1B). Basic features such as cell number and infected cell number were used to determine the “percentage of infected cells.” Several additional parameters such as size of cell and nucleus were also acquired that provided additional information to determine if the compound were toxic to cells was acquired (data not shown) but were not used in this study. Using these optimized conditions, a focused small molecule library of 840 serine/cysteine protease inhibitors from ChemDiv were screened against MP12 RVFV infection in HeLa cells.

Primary screening of the protease inhibitor library yielded 34 primary hits (Fig. 2), which could all be classified as one of four chemical structures (Fig. 3B). Interestingly, none of these four chemical scaffolds had been previously reported to possess antiviral activity. The 34 primary hits were further screened against the fully virulent RVFV ZH501 strain, VEEV, LASV, EBOV and MARV. These viruses represent four different viral families and thus would give us some indication if the hit compounds were broadly active. Many of the hit compounds were broadly active against all the viruses tested, albeit with different efficiencies. For example, compound C795-0925 had the highest activity against RVFV and VEEV, but exhibited moderate antiviral activity against EBOV, MARV, or LASV. Similarly, compound D011-2120 had high activity against EBOV, MARV, and VEEV, but moderate activity against RVFV or LASV (Fig. 3A). One compound from each chemical scaffold that showed broad spectrum antiviral activity (active against at least three of the viruses tested) were chosen for further study using a dose response curve assay against RVFV. Three of the four hit compounds had EC_50_ values that were below that of ribavirin, a drug commonly used to treat infections due to RNA viruses. To examine whether the compound's activities were specific to HeLa, we also tested them in the primary cell line SAEC. All of the hit compounds had activities similar in these cells as compared to those seen in HeLa cells; however, compound C795-0925 was less toxic in SAEC (CC_50_ 56.7 µM in HeLa compared to 110.6 µM in SAEC).

The virus life cycle is an orchestrated series of specific steps that include virus entry, viral RNA replication, transcription, followed by viral protein expression, and finally the assembly of the viral structural proteins and egress out of the cell. To determine the specific stage of virus life cycle targeted by the compounds, we first evaluated the kinetics of RVFV N, Gn and S-Gn expression (Fig. 4A) [28]. Cells treated with C795-0925and F694-1532 exhibited decreased levels of expression of the viral proteins N and Gn. In contrast, cells treated with D011-2120 and G202-0362 showed normal expression levels of N and Gn, similar to that seen in mock-treated virus infected cells. As expected, cells treated with C795-0925and F694-1532 also exhibited significantly reduced levels of surface expressed Gn (S-Gn), as fewer virions were being made during treatment with these compounds, thus fewer virions were getting to the cell surface. However, with compounds D011-2120 and G202-0362, cells exhibited normal levels of N and Gn, but greatly reduced levels of S-Gn, suggesting the inhibition was at a more downstream point in the viral life cycle, such as virus assembly or egress. Thus, we used time-of-addition assays to determine at which stage these compounds were acting. Since none of the compounds inhibited virus infection when added at the time of infection or 2 h prior to infection, we concluded that the compounds are not having an effect on virus entry (Fig. 4C). When time of compound addition was extended to 12.5 h PI, results suggest that D011-2120 and G202-0362 were affecting virus egress; whereas, C795-0925and F694-1532 were affecting viral RNA replication. We were further interested in using HCI-based analysis to define the mechanism of action used by D011-2120 and G202-0362 to inhibit virus egress.

RVFV glycoproteins localize to the Golgi, promote virus assembly, and bud into the Golgi lumen [14], [33]. Based on studies with other bunyaviruses, it is speculated that virus egress results from the budding of these virus-containing vesicles from the Golgi, trafficking through the cytoplasm, and their subsequent fusion with the plasma membrane, releasing the virus into the extracellular environment [15], [30], [33]. This is consistent with our data showing strong Gn staining in the Golgi at early time points (data not shown) and diffuse punctate stain throughout the cytoplasm at later time points (i.e., 10 h PI) (Fig. 5B). Presumably this represents RVFV-containing vesicles released from the Golgi and making their way to the cell surface as has been described for other bunyaviruses [22], [33]. These vesicles were not observed in cells stably expressing Gn alone (Fig. 6A), further supporting this idea. Given our observations, we wanted to determine if either G202-0362 or D011-2120 (compounds earlier shown to effect egress) affected viral trafficking from the Golgi to the cell surface. Interestingly, cells treated with G202-0362 showed Gn localized to the Golgi, but no virus could be seen in vesicles in the cytoplasm, suggesting that this compound was blocking the virus budding from Golgi and subsequent trafficking to the cell surface (Fig. 5). In contrast, D011-2120 appeared to completely disrupt the Golgi in a manner similar to nocodazole (i.e., by depolymerizing microtubules), as evidenced by co-localization of the Golgi marker, 1,4-galactosyltransferase, and Gn in a staining pattern similar to that seen with nocodazole (Fig. 5G–L). These data suggested that D011-2120, but not G202-0362, may be acting by disrupting cellular microtubules. Because we were interested in further defining the phenotypic alterations in the Golgi caused by treatment with D011-2120, we developed a HCI-based assay for this purpose. An algorithm using Columbus software was built that allowed classification of normal juxtanuclear Golgi complex from stacks of Golgi dispersed throughout the cytoplasm (Fig. 5B). The algorithm was applied to determine the potency of the compounds to determine the concentration of compound that gives 50% reduction in Golgi numbers (termed GC_50_) (Table 3). When these values were compared to EC_50_ values, it was evident that D011-2021 antiviral activity was related to its Golgi disruptive properties. Its GC_50_ was 38.2 µM, which was significantly lower than its EC_50_ (61.5 µM). Interestingly, while F694-1532 exhibited a relatively low GC_50_ (35.3 µM), it was still higher than its EC_50_ (20 µM). Likewise, as expected, F694-1532 and G202-0362 GC_50_ values were higher than their corresponding EC_50_ values, confirming that they also do not possess Golgi disruptive properties. We also examined the %GN_L_, which is the lowest percentage of Golgi number achievable at the compound's highest concentration. D011-2120 had the lowest %GN_L_ further suggesting this compound was an active Golgi disruptor. Because the staining pattern of cells treated with D011-2120 was similar to that seen with nocodazole treatment (Fig. 5), we hypothesized that D011-2120 was acting by a mechanism similar to that of nocodazole (i.e., depolymerization of microtubules). Because spindle fibers, which are made of microtubules, are an essential component involved in mitosis, and subsequently, cell division, a consequence of microtubule depolymerization is mitotic arrest. Consistent with the idea that D011-2120 exerts its effect by microtubule depolymerization, we showed that cells treated with this compound undergo mitotic arrest (Fig. 8A).

Collectively, our data suggest that the antiviral action of D011-2120 is by depolymerizing microtubules which disrupted virus trafficking of virions from the Golgi to the cell membrane during virus egress in RVFV infection. Microtubulin plays an important role in the life cycle of many viruses supporting virus entry, RNA replication, assembly and or egress (reviewed in [37])). The specific stage of the virus life cycle that is critically dependent on microtubulin defers between viruses and therefore it is possible that D011-2120 targeted virus entry or replication but not egress of other RNA viruses. Moreover microtubulin depolymerization induces mitotic arrest, and reduces cell viability in cells which can impair virus growth. This is apparent in the SI values of ≤1.2 derived from D011-2120 treatments in VEEV, EBOV, MARV or LASV infections, which meant that the compounds antiviral action could be due to compromised cell viability.

G202-0362 also affected virus egress, but it appears to do so by a different mechanism to that of D011-2120, namely by blocking virus budding from the *trans* Golgi. F694-1532 inhibits viral replication by an as yet unknown mechanism. This compound, however, also appeared to inhibit overall cellular gene expression and formed aggregates in the cell. Thus, while D011-2120 and F694-1532 may prove to have utility in treating other diseases, such as certain types of cancer, they are not attractive candidates for antiviral therapeutics. G202-0362 blocked virus budding from the Golgi, but did not disrupt the Golgi and did not alter any of the morphological features that we examined, including changes in cytoskeleton organization, cell or nucleus size, or Golgi or endoplasmic reticulum structure (data not shown). Moreover, this compound had no observable effects on cell division in uninfected cells at the highest concentration tested (200 µM). In addition this compound was effective against filoviruses and VEEV Likewise C795-0925 was highly effective against VEEV with EC_50_ = 10.2 µM and SI = 6.2. Thus, these two compounds may prove to be good candidates for antiviral drug development. In this study, we showed how HCI-based analysis could be used to screen for antiviral compounds effective against RVFV or other highly pathogenic RNA viruses and determine their mechanism of action.

## References

[1] Balkhy HH, Memish ZA (2003) Rift Valley fever: an uninvited zoonosis in the Arabian peninsula. Int J Antimicrob Agents 21: 153–157. 12615379 10.1016/s0924-8579(02)00295-9

[2] Daubney R, Hudson JR, Garnham PC (1931) Enzootic hepatitis or rift valley fever. An undescribed virus disease of sheep cattle and man from east africa. The Journal of Pathology and Bacteriology 34: 545–579.

[3] Laughlin LW, Meegan JM, Strausbaugh LJ, Morens DM, Watten RH (1979) Epidemic Rift Valley fever in Egypt: observations of the spectrum of human illness. Trans R Soc Trop Med Hyg 73: 630–633. 575446 10.1016/0035-9203(79)90006-3

[4] Madani TA, Al-Mazrou YY, Al-Jeffri MH, Mishkhas AA, Al-Rabeah AM, et al (2003) Rift Valley fever epidemic in Saudi Arabia: epidemiological, clinical, and laboratory characteristics. Clin Infect Dis 37: 1084–1092. 14523773 10.1086/378747

[5] Brown JL, Dominik JW, Morrissey RL (1981) Respiratory infectivity of a recently isolated Egyptian strain of Rift Valley fever virus. Infect Immun 33: 848–853. 7287187 10.1128/iai.33.3.848-853.1981 PMC350789

[6] Patrican LA, Bailey CL (1989) Ingestion of immune bloodmeals and infection of Aedes fowleri, Aedes mcintoshi, and Culex pipiens with Rift Valley fever virus. Am J Trop Med Hyg 40: 534–540. 2729509 10.4269/ajtmh.1989.40.534

[7] Bray M, Murphy FA (2007) Filovirus research: knowledge expands to meet a growing threat. J Infect Dis 196 Suppl 2: S438–443. 17940981 10.1086/520552

[8] Yun NE, Walker DH (2012) Pathogenesis of Lassa fever. Viruses 4: 2031–2048. 23202452 10.3390/v4102031 PMC3497040

[9] Quiroz E, Aguilar PV, Cisneros J, Tesh RB, Weaver SC (2009) Venezuelan equine encephalitis in Panama: fatal endemic disease and genetic diversity of etiologic viral strains. PLoS Negl Trop Dis 3: e472. 19564908 10.1371/journal.pntd.0000472 PMC2697379

[10] Bickle M (2010) The beautiful cell: high-content screening in drug discovery. Anal Bioanal Chem 398: 219–226. 20577725 10.1007/s00216-010-3788-3

[11] Struthers JK, Swanepoel R, Shepherd SP (1984) Protein synthesis in Rift Valley fever virus-infected cells. Virology 134: 118–124. 6710870 10.1016/0042-6822(84)90277-0

[12] de Boer SM, Kortekaas J, Spel L, Rottier PJ, Moormann RJ, et al (2012) Acid-activated structural reorganization of the Rift Valley fever virus Gc fusion protein. J Virol 86: 13642–13652. 23035232 10.1128/JVI.01973-12 PMC3503025

[13] Pettersson RF, Melin L (1996) Synthesis, assembly, and intracellular transport of Bunyaviridae membrane proteins. In: Elliott RM, editor. The Bunyaviridae. New York, NY: Plenum Press pp. 159–188.

[14] Carnec X, Ermonval M, Kreher F, Flamand M, Bouloy M (2014) Role of the cytosolic tails of Rift Valley fever virus envelope glycoproteins in viral morphogenesis. Virology 448: 1–14. 24314631 10.1016/j.virol.2013.09.023

[15] Caplen H, Peters CJ, Bishop DH (1985) Mutagen-directed attenuation of Rift Valley fever virus as a method for vaccine development. J Gen Virol 66 Pt 10: 2271–2277. 4045430 10.1099/0022-1317-66-10-2271

[16] Habjan M, Pichlmair A, Elliott RM, Overby AK, Glatter T, et al (2009) NSs protein of rift valley fever virus induces the specific degradation of the double-stranded RNA-dependent protein kinase. J Virol 83: 4365–4375. 19211744 10.1128/JVI.02148-08 PMC2668506

[17] Filone CM, Hanna SL, Caino MC, Bambina S, Doms RW, et al (2010) Rift valley fever virus infection of human cells and insect hosts is promoted by protein kinase C epsilon. PLoS One 5: e15483. 21124804 10.1371/journal.pone.0015483 PMC2991366

[18] Shum D, Smith JL, Hirsch AJ, Bhinder B, Radu C, et al (2010) High-content assay to identify inhibitors of dengue virus infection. Assay Drug Dev Technol 8: 553–570. 20973722 10.1089/adt.2010.0321 PMC2962577

[19] Cruz DJ, Koishi AC, Taniguchi JB, Li X, Milan Bonotto R, et al (2013) High content screening of a kinase-focused library reveals compounds broadly-active against dengue viruses. PLoS Negl Trop Dis 7: e2073. 23437413 10.1371/journal.pntd.0002073 PMC3578765

[20] Turk B (2006) Targeting proteases: successes, failures and future prospects. Nat Rev Drug Discov 5: 785–799. 16955069 10.1038/nrd2092

[21] Ikegami T, Narayanan K, Won S, Kamitani W, Peters CJ, et al (2009) Rift Valley fever virus NSs protein promotes post-transcriptional downregulation of protein kinase PKR and inhibits eIF2alpha phosphorylation. PLoS Pathog 5: e1000287. 19197350 10.1371/journal.ppat.1000287 PMC2629125

[22] Morrill JC, Ikegami T, Yoshikawa-Iwata N, Lokugamage N, Won S, et al (2010) Rapid accumulation of virulent rift valley Fever virus in mice from an attenuated virus carrying a single nucleotide substitution in the m RNA. PLoS One 5: e9986. 20376320 10.1371/journal.pone.0009986 PMC2848673

[23] Lang SA, Kozyukov AV, Balakin KV, Skorenko AV, Ivashchenko AA, et al (2002) Classification scheme for the design of serine protease targeted compound libraries. J Comput Aided Mol Des 16: 803–807. 12825792 10.1023/a:1023832728547

[24] Drosten C, Gottig S, Schilling S, Asper M, Panning M, et al (2002) Rapid detection and quantification of RNA of Ebola and Marburg viruses, Lassa virus, Crimean-Congo hemorrhagic fever virus, Rift Valley fever virus, dengue virus, and yellow fever virus by real-time reverse transcription-PCR. J Clin Microbiol 40: 2323–2330. 12089242 10.1128/JCM.40.7.2323-2330.2002 PMC120575

[25] Livak KJ, Schmittgen TD (2001) Analysis of relative gene expression data using real-time quantitative PCR and the 2(−Delta Delta C(T)) Method. Methods 25: 402–408. 11846609 10.1006/meth.2001.1262

[26] Ikegami T, Won S, Peters CJ, Makino S (2006) Rescue of infectious rift valley fever virus entirely from cDNA, analysis of virus lacking the NSs gene, and expression of a foreign gene. J Virol 80: 2933–2940. 16501102 10.1128/JVI.80.6.2933-2940.2006 PMC1395455

[27] Zhang JH, Chung TD, Oldenburg KR (1999) A Simple Statistical Parameter for Use in Evaluation and Validation of High Throughput Screening Assays. J Biomol Screen 4: 67–73. 10838414 10.1177/108705719900400206

[28] Mudhasani R, Tran JP, Retterer C, Radoshitzky SR, Kota KP, et al (2013) IFITM-2 and IFITM-3 but not IFITM-1 restrict Rift Valley fever virus. J Virol 87: 8451–8464. 23720721 10.1128/JVI.03382-12 PMC3719792

[29] Garcia S, Crance JM, Billecocq A, Peinnequin A, Jouan A, et al (2001) Quantitative real-time PCR detection of Rift Valley fever virus and its application to evaluation of antiviral compounds. J Clin Microbiol 39: 4456–4461. 11724861 10.1128/JCM.39.12.4456-4461.2001 PMC88565

[30] Hepojoki J, Strandin T, Wang H, Vapalahti O, Vaheri A, et al (2010) Cytoplasmic tails of hantavirus glycoproteins interact with the nucleocapsid protein. J Gen Virol 91: 2341–2350. 20444994 10.1099/vir.0.021006-0

[31] Rogalski AA, Singer SJ (1984) Associations of elements of the Golgi apparatus with microtubules. J Cell Biol 99: 1092–1100. 6381504 10.1083/jcb.99.3.1092 PMC2113400

[32] Doms RW, Russ G, Yewdell JW (1989) Brefeldin A redistributes resident and itinerant Golgi proteins to the endoplasmic reticulum. J Cell Biol 109: 61–72. 2745557 10.1083/jcb.109.1.61 PMC2115463

[33] Strandin T, Hepojoki J, Vaheri A (2013) Cytoplasmic tails of bunyavirus Gn glycoproteins-Could they act as matrix protein surrogates? Virology 437: 73–80. 23357734 10.1016/j.virol.2013.01.001

[34] De Brabander MJ, Van de Veire RM, Aerts FE, Borgers M, Janssen PA (1976) The effects of methyl (5-(2-thienylcarbonyl)-1H-benzimidazol-2-yl) carbamate, (R 17934; NSC 238159), a new synthetic antitumoral drug interfering with microtubules, on mammalian cells cultured in vitro. Cancer Res 36: 905–916. 766963

[35] Jordan MA, Thrower D, Wilson L (1992) Effects of vinblastine, podophyllotoxin and nocodazole on mitotic spindles. Implications for the role of microtubule dynamics in mitosis. J Cell Sci 102 Pt 3: 401–416. 1506423 10.1242/jcs.102.3.401

[36] Hendzel MJ, Wei Y, Mancini MA, Van Hooser A, Ranalli T, et al (1997) Mitosis-specific phosphorylation of histone H3 initiates primarily within pericentromeric heterochromatin during G2 and spreads in an ordered fashion coincident with mitotic chromosome condensation. Chromosoma 106: 348–360. 9362543 10.1007/s004120050256

[37] Radtke K, Dohner K, Sodeik B (2006) Viral interactions with the cytoskeleton: a hitchhiker's guide to the cell. Cell Microbiol 8: 387–400. 16469052 10.1111/j.1462-5822.2005.00679.x

